# eDNA confirms lower trophic interactions help to modulate population outbreaks of the notorious crown-of-thorns sea star

**DOI:** 10.1073/pnas.2424560122

**Published:** 2025-03-10

**Authors:** Kennedy Wolfe, Amelia A. Desbiens, Frances Patel, Sarah Kwong, Eric Fisher, Peter J. Mumby, Sven Uthicke

**Affiliations:** ^a^School of the Environment, The University of Queensland, Brisbane, QLD 4072, Australia; ^b^Marine Biology and Ecology Consultant, Geeveston, TAS 7116, Australia; ^c^Commonwealth Scientific and Industrial Research Organisation Environment, Brisbane, QLD 4072, Australia; ^d^Australian Institute of Marine Science, Townsville, QLD 4810, Australia; ^e^College of Science and Engineering, James Cook University, Townsville, QLD 4811, Australia; ^f^Great Barrier Reef Biology, Experience Co., Cairns, QLD 4870, Australia

**Keywords:** juvenile, structural equation model, predation, molecular, pest species

## Abstract

Variability in predator–prey interactions can modulate population dynamics with impacts scalable to entire ecosystems. As notorious corallivores, crown-of-thorns sea stars (CoTS; *Acanthaster* spp.) have caused extensive losses of coral habitat during unexplained population outbreaks across the Indo-Pacific. While predation of adult CoTS may help to suppress their outbreaks, it does not sufficiently explain their profound boom-bust dynamics and so remains equivocal. Factors influencing early postsettlement mortality are generally more impactful on population size, thus lower trophic interactions involving juvenile CoTS may better contribute to outbreak prevention. We evaluated the impact of key predatory decapods that interact with juvenile CoTS in their coral rubble nursery before they emerge as destructive corallivores. Decapod density was influenced by habitat complexity and varied regionally, inverse to spatial trends in CoTS outbreaks on the Great Barrier Reef. Using eDNA gut content analysis, we confirmed seven species (~12% of individuals) of wild-caught decapod, collected from two reefs separated by >1,000 km, as CoTS predators. Owing to spatial variation in predator abundance and community structure, we estimated potential (previous aquarium experiments) and realized (eDNA results here) rates of CoTS consumption were ~3-fold and ~1.6-fold lower, respectively, in outbreak hotspots. Through combination of field and molecular techniques, we demonstrated the appreciable impact of cryptic predators on early population success of this nuisance species, which expands our knowledge of CoTS outbreaks, pest species management, and reef conservation. Resolving predator–prey interactions at lower levels of the ecosystem can be crucial to understanding broader ecological outcomes.

## Significance

The crown-of-thorns sea star (CoTS) is a nuisance species owing to its mass consumption of coral during destructive unexplained population outbreaks. Variability in predation of CoTS may help to modulate outbreaks, but hypotheses have overlooked the impact of cryptic predators on juvenile CoTS in their rubble nursery before they transition into large corallivores. We found fewer cryptic predators in areas prone to CoTS outbreaks and developed an eDNA method to detect the realized impact of this lower-trophic interaction in a diversity of wild-caught decapods separated by >1,000 km of reef. Outcomes challenge the view that higher-order species chiefly regulate CoTS outbreaks and demonstrate that predator–prey interactions at lower levels of the ecosystem can scale to alter trophodynamics with key conservation benefits.

Characterizing how species interactions shape trophic outcomes is a principal goal in ecology, as changes in the population size of a single species can scale to affect entire communities (1) and the social, cultural, and economic values and stability of ecosystems (2, 3). Pest species provide hallmark examples of this, whereby the disproportionate success of one species can have cascading impacts on community structure, food webs, and ecosystem processes (4). In an age of applied science, determining what drives the success of species with putatively deleterious ecological influence must be resolved to help minimize their impacts against a background of amplifying local and global stressors in the Anthropocene (4, 5).

One of the most notorious nuisance species in the tropical Indo-Pacific is the crown-of-thorns sea star (CoTS; *Acanthaster* spp.), a common native asteroid group of at least five species (6, 7). The propensity of CoTS to exhibit high-density boom-bust population outbreaks has generated decades of conservation concern and investment (8). As large corallivores, CoTS outbreaks have been attributed to substantial declines in coral cover with ecological and economic impacts across its range (9–11). Identification of the proximal cause(s) of CoTS outbreaks is crucial to inform conservation and management.

Several primary hypotheses have been used to explain CoTS outbreaks, though they remain widely debated (8, 12). The predator removal hypothesis postulates that overfishing of higher-order predators (i.e., reef fishes) has alleviated top–down control of CoTS with great ecological consequence (13). Despite the growing number of fishes considered predators of CoTS (14), the relatively small reduction in adult population density through differential predation seems insufficient to explain the boom-bust nature of CoTS (8), likely working to suppress rather than prevent outbreaks. CoTS outbreaks must almost certainly involve massive pulses of successful recruitment, which suggests that variation in larval and/or early postsettlement success would be most impactful on CoTS population size and outbreak generation (8, 15).

In marine systems, even small variations in predator-induced mortality during early life history stages can accumulate to have disproportionate (“Allee”) effects on population size (16, 17). For CoTS, gametes and larvae can experience high predation pressure from planktivores (18, 19) while mortality of newly settled juveniles may reduce populations by >90% within weeks of development (20–22). Variability in predation of juvenile CoTS in its coral rubble nursery has great potential to be a proximal cause of CoTS population outbreaks (22–24), making the drivers of this variation of great interest. However, predation in cryptic habitats is difficult to observe and quantify using traditional survey methods (25), such as video assays (e.g., ref. 26). Whether predation of juvenile CoTS as observed in aquaria (18, 27, 28) substantially varies owing to cryptic community abundance and composition in the wild remains undetected.

We approached this problem using two methods to estimate predation of juvenile CoTS in rubble: 1) potential predation was quantified by combining rates of consumption from experimental feeding trials (27) with spatial data on predator density and 2) realized predation was quantified through the incidence of CoTS consumption detected through eDNA gut content analysis of wild-caught predators. As a data-poor group, field surveys were first conducted to characterize the density, distribution, and habitat associations of select predatory decapods (see ref. 27) across a wide latitudinal gradient (>1,000 km) of the Great Barrier Reef (GBR), Australia. Predators were contrast on reefs with high (northern and central GBR) and low (southern GBR) CoTS outbreak histories (12, 29). Bayesian structural equation modeling (SEM) was used to assess hierarchical local- and regional-scale predictors of predators to inform reef management and CoTS surveillance. Then, predators were collected from the southern and northern GBR aiming to confirm this cryptic predator–prey interaction using eDNA methods established here with CoTS-specific primers (14, 30–33), adding to the arsenal of molecular techniques in the early detection and management of pest species (34, 35).

Through the combination of field and molecular approaches, we extrapolated potential and realized rates of predation of juvenile CoTS by key cryptic decapods and discuss their relevance to outbreak generation. As management of CoTS currently depends on manual harvest of large conspicuous individuals (36), this study on cryptic predators that interact with juvenile CoTS before they emerge as destructive corallivores is of great importance to understanding natural mechanisms of suppressing and preventing outbreaks to expand the management toolbox of this impactful species. Knowledge gained highlights the importance of considering lower trophic interactions in research and management involving CoTS and in coral reef ecology more broadly.

## Results

### Characterizing Cryptic Predators.

**Fig. 1.**
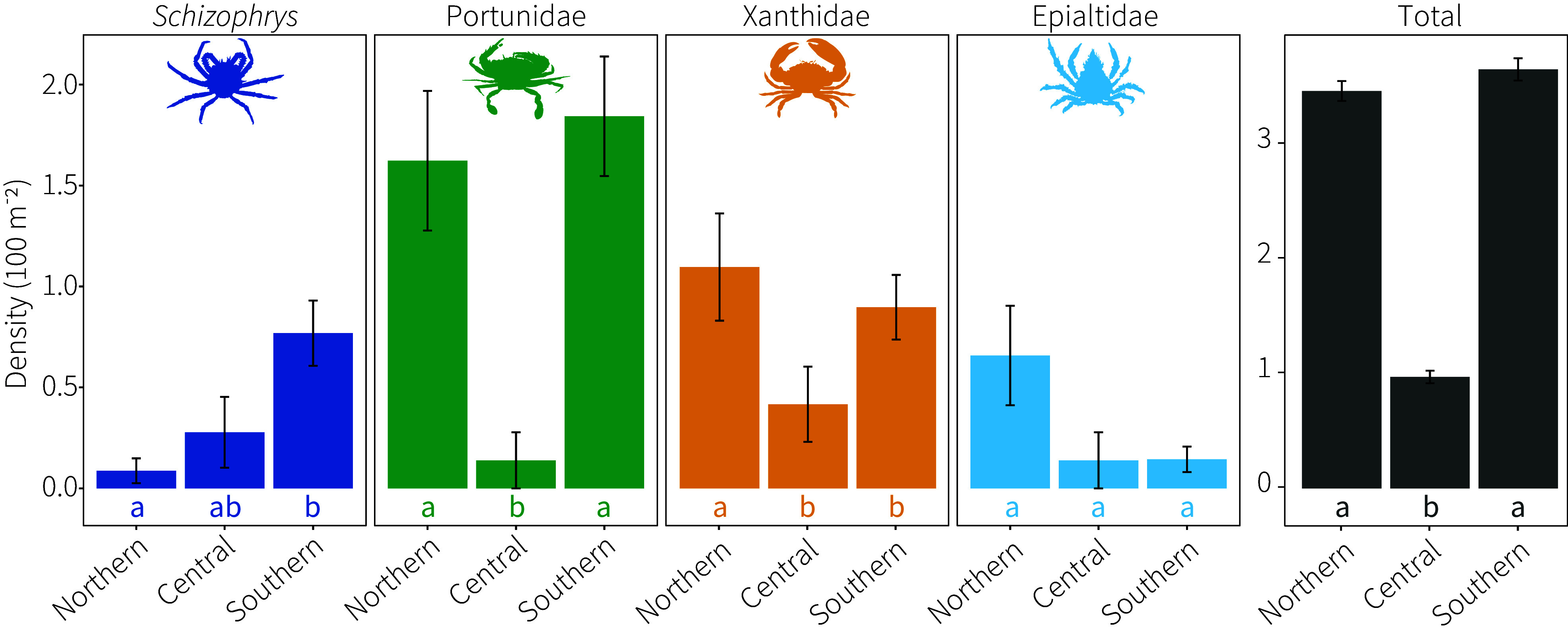
Mean (± SE) density of key predators of juvenile CoTS in rubble of the northern (n = 57), central (n = 6), and southern (n = 52) GBR. Tukey’s post hoc: letters that are the same do not differ.

Predator density was surveyed in transects in the southern (n = 52), central (n = 6), and northern (n = 57) GBR (*SI Appendix*, Fig. S1). Total predator density was higher in the southern and northern GBR than in the central region, and species composition differed (Fig. 1 and Table 1). Mean density of *Schizophrys aspera*, a decorator crab with the highest rates of CoTS consumption in aquaria (27), was highest in the south with the fewest found on reefs in the north (Fig. 1 and *SI Appendix*, Fig. S2). Additionally, *S. aspera* were larger in size (*P* = 0.002) in the south (22.6 ± 0.8 mm) compared to the central (16.4 ± 4.7 mm) and northern (16.8 ± 1.2 mm) GBR (*SI Appendix*, Fig. S3). Portunid crabs were most abundant in the north and south, while few were found in the central GBR (Fig. 1). Xanthid crabs were higher in density in the northern GBR than in the central and south, while epialtids did not differ (Fig. 1).

**Table 1.** Summary *glmmTMB* results on the density of key predators of juvenile CoTS in rubble of the northern, central, and southern GBR

Species	Chi-sq	df	Pr(>Chi-sq)
*S. aspera*	13.5	2	**0.001**
Portunidae	15.9	2	**<0.001**
Xanthidae	24.3	2	**<0.001**
Epialtidae	3.5	2	0.173
Total	12.1	2	**0.002**

Significant values (p < 0.05) in bold.

**Fig. 2.**
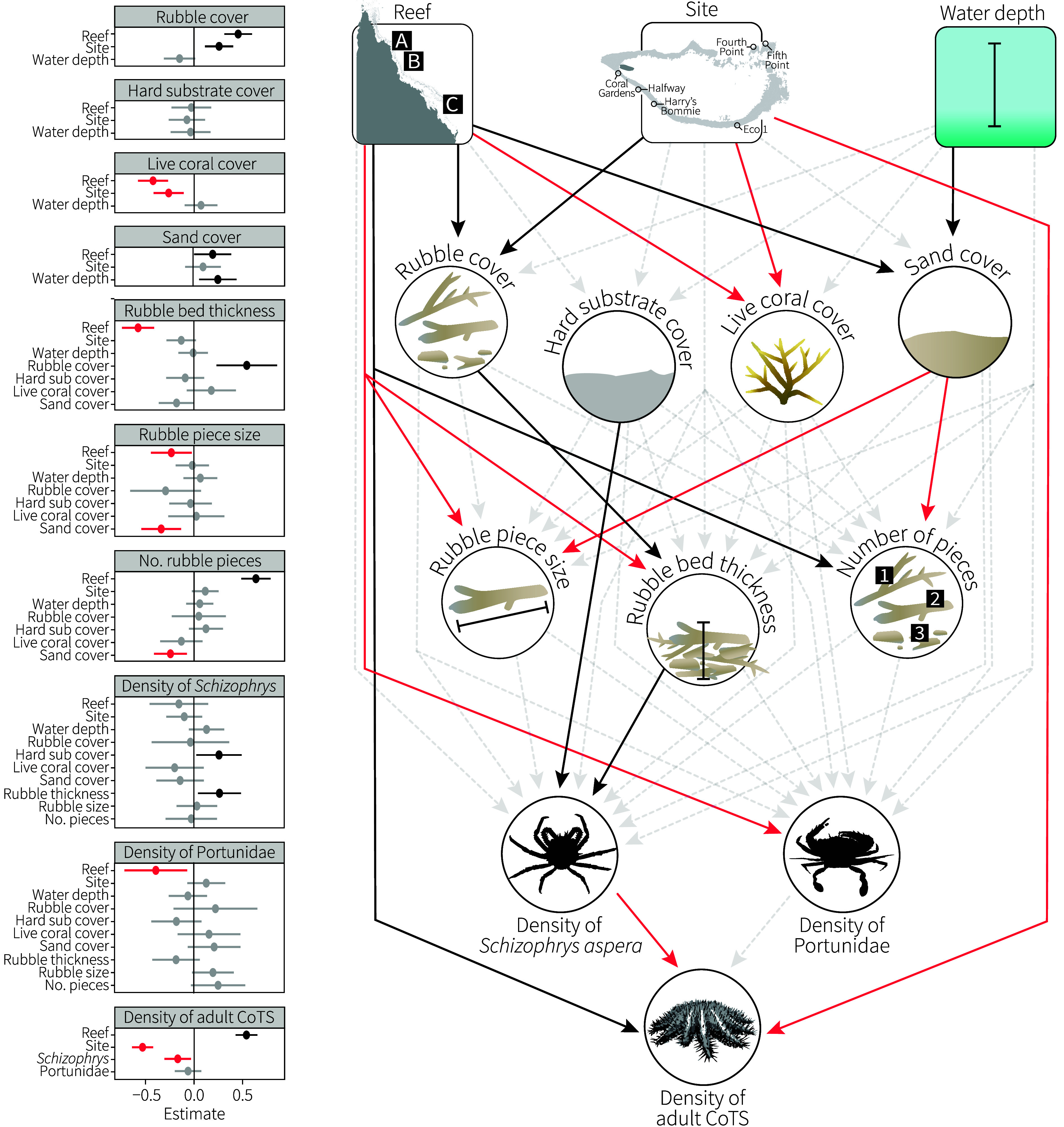
Path diagram of Bayesian SEM describing the local- and regional-scale drivers of the density of *S. aspera* and the Portunidae and their effects on adult CoTS. Solid black arrows indicate significant positive pathways, solid red arrows indicate significant negative pathways, and gray dotted arrows show nonsignificant pathways. Plots show significant positive (black) and negative (red) scaled estimate and error results of each interaction.

A Bayesian SEM was used to interrogate local- and regional-scale drivers in the relationships between habitat characteristics, cryptic predators, and adult CoTS (CoTS Control Program; *SI Appendix*, Table S2). Regional drivers (reef, site, and water depth) influenced the benthic cover of rubble, live coral, and sand, including an increase in sand with water depth (Fig. 2). Rubble cover had a positive effect on rubble bed thickness, which, along with the hard substrate, was a positive driver of the density of *S. aspera* (Fig. 2). This relationship continued with a negative influence of *S. aspera* on adult CoTS density (Fig. 2). Cover of sand had a negative influence on the size and number of rubble pieces, which had marginally insignificant effects on the density of portunid crabs (Fig. 2). Portunid density varied more broadly by reef and had no significant relationship with adult CoTS density (Fig. 2). Along with *S. aspera*, regional drivers of reef and site influenced CoTS. Live coral had no influence on rubble metrics nor rubble predators in this assessment.

### Molecular eDNA Detection.

**Fig. 3.**
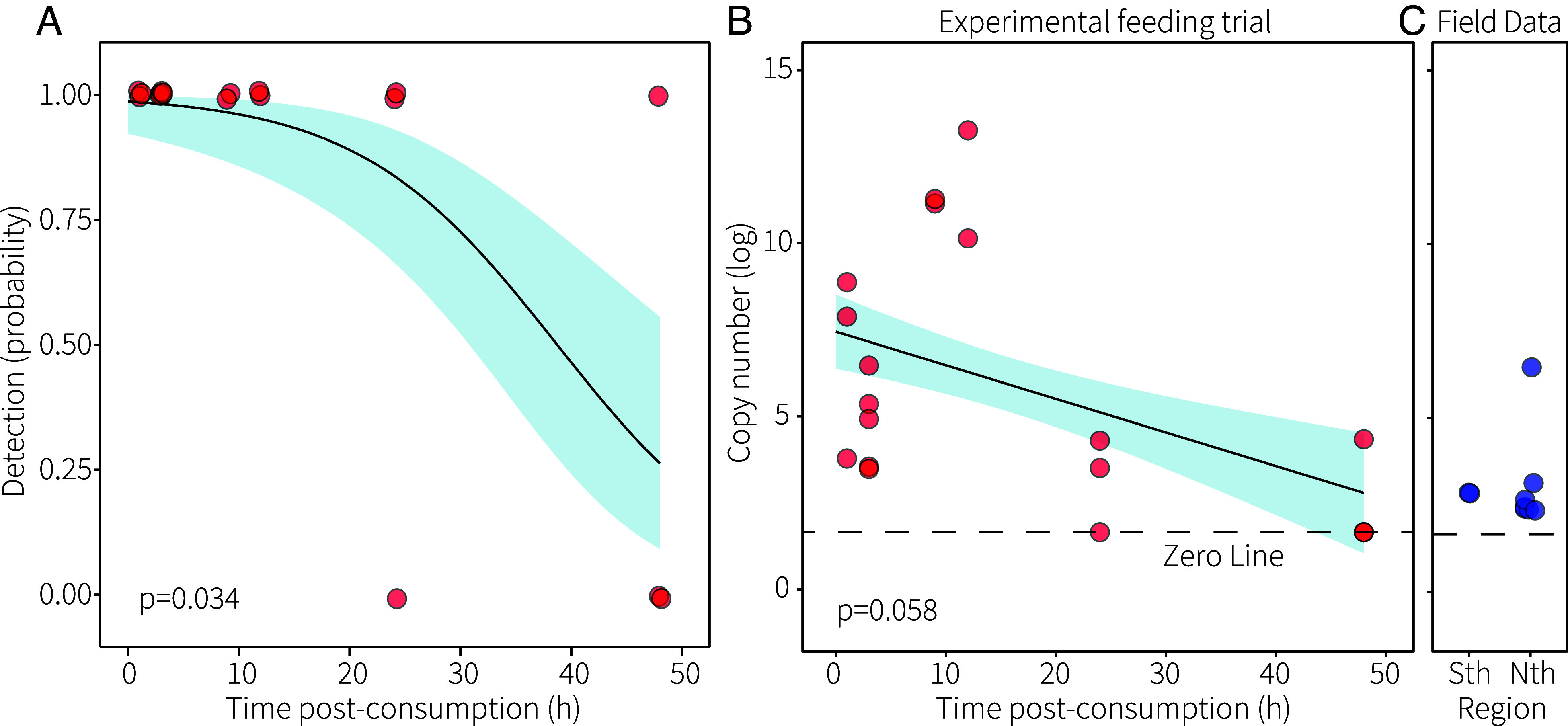
(*A*) Probability of detection and (*B*) DNA copy number (log-scale) within 48 h postconsumption of a single juvenile in timed experimental feeding trials, and (*C*) DNA copy numbers in field-positive cryptic predators in the southern (Sth) and northern (Nth) GBR.

Experimental feeding trials were conducted to determine sensitivity of the eDNA method to CoTS DNA detection in three digestive tissues of cryptic predators (stomach, midgut, and abdomen). All specimens from experimental feeding trials tested positive for CoTS DNA at 1, 3, 9, and 12 h postconsumption (Fig. 3*A*). Probability of detection was 97% within 12 h and declined thereafter to ~33% at 48 h, the longest digestion time evaluated (Fig. 3*A*). DNA copy numbers (averaged over the three tissue types) in specimens from experimental feeding trials were highest at 9 to 12 h postconsumption and declined over time (Fig. 3*B*). Subsequently, we collected twelve *S. aspera* (southern GBR) and 63 cryptic predators (northern GBR) at the time juvenile CoTS are available at the appropriate size in rubble on the GBR [~March; (37)]. 17% (2 of 12) of *S. aspera* from the southern GBR and 11% (7 of 63) of cryptic predators from the northern GBR tested positive for CoTS DNA (*SI Appendix*, Table S3). In the north, CoTS DNA was detected in six species of decapod from the Epialtidae, Portunidae, and Xanthidae, but in zero (of five) *S. aspera* (*SI Appendix*, Table S3). DNA copy numbers indicated wild-type predators mostly likely interacted with CoTS within the most conservative timeframes (24 to 48 h), though high copy numbers for one predator (Xanthidae; *Cyclodius ungulatus*) suggests that its interaction was sooner (Fig. 3*C*).

### Scalable Impact of Cryptic Predators.

**Fig. 4.**
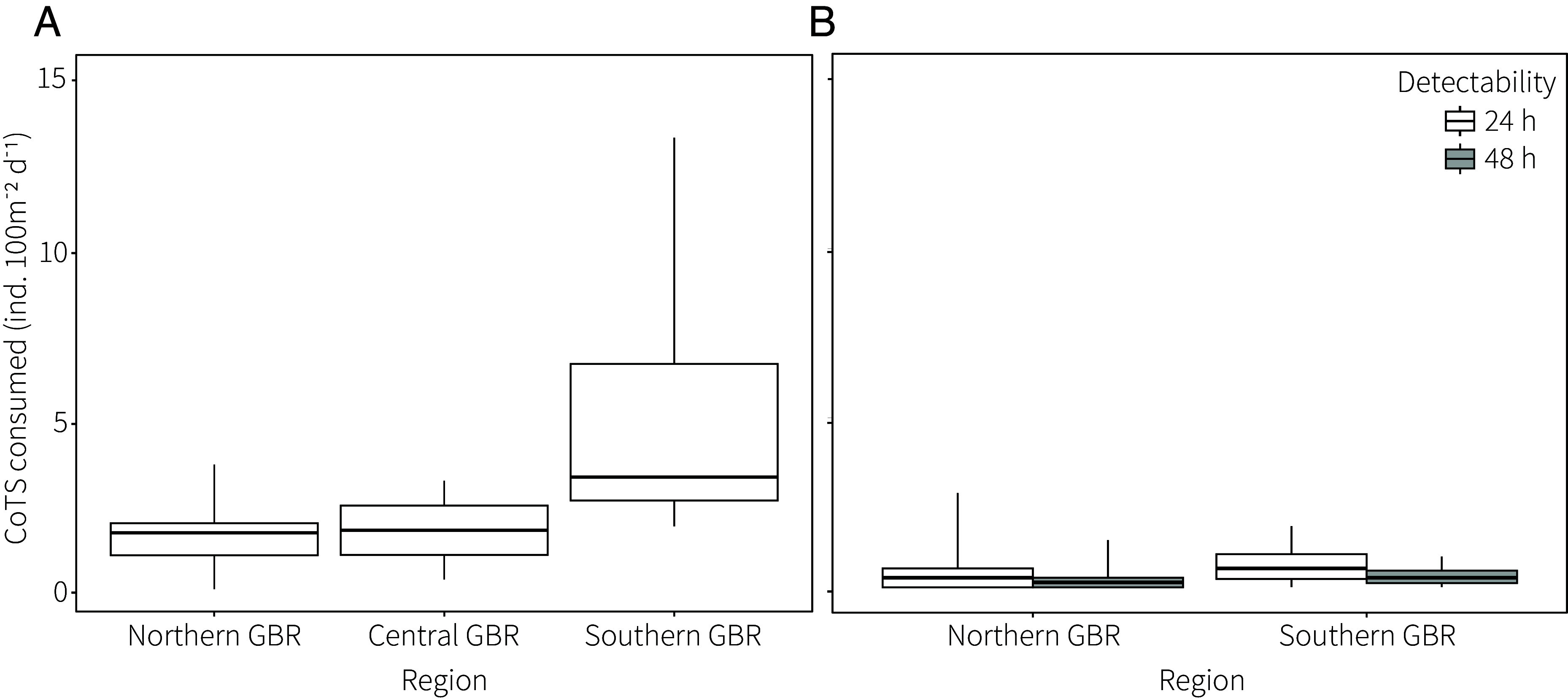
Extrapolated (*A*) potential and (*B*) realized rates of daily predation of CoTS (100 m^−2^) by cryptic decapods on the GBR. Realized rates derived from assumed DNA detection sensitivity 24 and 48 h postconsumption (*Materials and Methods*). Boxes represent the interquartile ranges (0.25 and 0.75), the horizontal line is the median, and the whiskers are the data range.

Impact of cryptic predators was contrast in outbreak (northern and central) and nonoutbreak (southern) prone regions of the GBR. Daily rates of potential predation were extrapolated based on experimentally derived rates of predation of CoTS (27) and cryptic predator density (this study). Average predation potential was ~3-fold higher in the southern GBR (5.4 ind. 100 m^−2^ d^−1^) than in central (1.9 ind. 100 m^−2^ d^−1^) and northern (1.7 ind.100 m^−2^ d^−1^) regions (Fig. 4*A*), inverse to spatial trends in CoTS outbreaks (29). This difference was driven by the combined high density and feeding rates of *S. aspera* in the south, with a maximum predation potential of 13.4 ind. 100 m^−2^ d^−1^. Realized rates of predation, calculated based on positive CoTS DNA detection in wild-type specimens, were comparably lower (Fig. 4*B*). Assuming detection sensitivity is 24 to 48 h, as indicated by the proportion of positive samples and DNA copy numbers (Fig. 3 *A*–*C*), realized predation rates of CoTS were 0.6 to 0.3 and 0.4 to 0.2 ind. 100 m^−2^ d^−1^ in the southern and northern GBR, respectively (Fig. 4*B*). Realized predation was ~1.6 times higher in the south than the north (Fig. 4*B*), with a greater margin between realized and potential predation.

## Discussion

Our study provides some of the first available data on the density and distribution of key cryptic predators of CoTS across the GBR through the use of an effective, nondestructive survey method in rubble. We found the density and community structure of cryptic decapods differed owing to habitat complexity and reef region. Specifically, spatial variability in the density of *S. aspera* and its association with rubble may be a bioindicator of adult CoTS density and thus outbreak potential, supporting experimental (27) and local-scale (38) evidence. Calculated potential and realized rates of predation of CoTS were consequently inverse to regional trends in CoTS outbreaks on the GBR whereby; 1) reefs with low densities of cryptic predators may be susceptible to more frequent and severe outbreaks, as experienced at Moore Reef (39, 40); 2) cryptic communities with fewer *S. aspera* may not contribute sufficiently to rates of predation to curb juvenile cohort progression, as experienced at Lizard Island (41, 42); and 3) cryptic communities with higher densities of *S. aspera* could generate a ~3-fold greater potential and ~1.6-fold greater realized impact on CoTS populations, which may appreciably impact adult CoTS density, as experienced at Heron Island (38). It seems of great interest to determine whether these patterns are maintained on additional reefs and regions to those characterized here.

Visual detection of newly settled juvenile CoTS is extremely challenging (37), yet we detected CoTS DNA in 12% of cryptic predators collected on reefs >1,000 km apart during the juvenile recruitment period on the GBR. This outcome confirms 1) the frequent interaction of cryptic predators with CoTS in nature, 2) the viability of identifying novel CoTS predators using this eDNA method, and 3) that cryptic predators may be useful bioindicators of CoTS populations. High detection (17% of individuals) of CoTS DNA in *S. aspera* in the southern GBR confirms the capacity of this species to interact with CoTS in nature, an observation previously restricted to controlled aquarium experiments (27). However, zero (of five) *S. aspera* were found with CoTS DNA in the north, highlighting the need for greater sampling to address the variable contribution of these predators to CoTS mortality across the GBR. That six other species were found with CoTS DNA in the north may offer some redundancy to this trophic interaction, though possibly with lower total impact on CoTS. Notably, we could not differentiate whether predators consumed one or multiple juveniles, caused injury through partial predation, or interacted with CoTS otherwise. Further data are needed to determine how DNA copy numbers reflect varying degrees of predation (e.g., ref. 27), multiple prey interactions, and the precise time at which CoTS DNA is undetectable, so that copy numbers can be used to predict juvenile density (43).

Our extrapolations showed that the cryptic predator communities surveyed could potentially consume 5.4 and 1.7 ind. 100 m^−2^ d^−1^ in the southern and northern GBR, respectively. Realized predation rates were markedly lower, suggesting major limiting factors (e.g., prey availability, habitat complexity, competition) to how this interaction plays out in nature, perhaps especially in the southern GBR where the margin between realized and potential predation was twice that of the north. Indeed, high densities of juvenile CoTS as required to generate outbreaks would result in greater probability of encounter by predators, closing the gap between realized and potential predation. Accumulation or “armies” of juveniles in rubble (44) would likely saturate cryptic predators to seed outbreaks (45). However, the negative relationship detected here between adult CoTS and *S. aspera* supports the hypothesis that variation in cryptic community structure contributes to outbreak suppression on the GBR. Resolving why CoTS consumption rates vary between regions (e.g., habitat complexity, predator community structure, prey alternatives, and anthropogenic stressors) should be a major future research direction.

Our results demonstrate that the degree of impact of cryptic predators on CoTS is likely constrained by benthic habitat structure. Predator density varied based on rubble bed characteristics including total rubble cover and bed thickness, with *S. aspera* preferring deep rubble interstices as found at Heron Island. Rubble pieces were smaller and beds tended to be thinner in profile on reefs in the central and northern GBR, where complete disintegration of dead coral is rapid (46). Juvenile CoTS may be primed to survive in these rubble types where they are exposed to fewer high-impact predators. Additional, thoughtfully linked surveys are required to address whether cryptic predators and juvenile CoTS are responding to the same hierarchical environmental drivers in rubble, and the ontogenetic timeframes in which CoTS are susceptible to cryptic predation. For now, research and monitoring programs may benefit from considering rubble bed thickness as a key benthic predictor *S. aspera* and thus CoTS outbreak potential.

We present a different perspective for CoTS outbreaks whereby the hierarchical effects of rubble structure on cryptic predators may generate scalable impacts on the success of a destructive corallivore, supporting the recently described “degraded reef hypothesis” (45). If decapods are lacking in specific rubble types, alleviated top–down control of juveniles may help to seed outbreaks. Subsequent impacts to corals would exacerbate rubble generation (47) providing more habitat for juvenile CoTS and/or cryptic predators (45). This negative feedback loop is important to evaluate, especially if rubble becomes a more prevalent dead foundation species in the Anthropocene (48–51).

Management of predators has never been a component of CoTS population control on the GBR (52), as causal links between CoTS and higher-order predators (e.g., reef fishes) are elusive (8). Following our findings, we posit a shift from the “predator removal hypothesis” to explain outbreaks to the “variable predator hypothesis” to more holistically encapsulate varying degrees of higher- and lower-order predation-based regulation of CoTS across their multiple, distinct life stages. As cryptic predators may be an important natural mechanism of control before CoTS are visually detectable, management innovations that consider juveniles in rubble are needed to augment the time- and labor-intensive manual harvest of large conspicuous adults (36, 53). Impacts facing cryptic predators are largely unknown, but this knowledge is crucial to determine whether their populations are at risk and require protection to manage their role in outbreak suppression and/or prevention. If cryptic predator densities are depleted in certain regions, *S. aspera* may be considered a candidate for biocontrol, a widely applied tool in pest species management (54–56) that has been suggested for CoTS through use of species-specific peptides and attractants (57, 58) and restocking of *Charonia tritonis* (59, 60). We strongly advise that much research is required before considering seeding populations as a management tool, which can cause negative impacts without rigorous interrogation (54). The demonstrated importance of *S. aspera* as a key CoTS predator promotes further inquiry on its natural distribution and taxonomy (61), and ecological interactions with other species (62), which are required to inform the appropriateness of its use in a biocontrol context.

Our data indicate that CoTS populations may be, at least in part, controlled by predator community type and/or the environmental and disturbance history that affects reef and rubble characteristics and thus predator density. This adds to the knowledge base that habitat complexity and community structure can shape lower trophic interactions involving decapods (63, 64) with consequent impacts that involve pest species (65, 66). Use of eDNA in ecosystem-based management and surveillance is growing exponentially, including in the early detection of invasive species in marine and terrestrial ecosystems (34, 35). Our study contributes to this arsenal to help tackle the CoTS problem using eDNA (14, 30–33). Coupling molecular techniques with traditional survey methods, as done here, provides a valuable tool to evaluate complex and cryptic lower trophic interactions to inform management and help resolve a major issue facing coral reefs. Lower trophic interactions may indeed have appreciable impacts on CoTS populations with potential knock-on effects on the ecosystem state.

## Materials and Methods

### Site Selection.

Field surveys were conducted at select reefs in the southern (Heron Island, March 2023), central (Moore Reef, June 2023), and northern (Lizard Island, March 2024) GBR, Australia (*SI Appendix*, Fig. S1). Site selection was informed by CoTS outbreak history, rubble availability determined through site reconnaissance, and eDNA studies involving CoTS larvae and recruits (32, 33). Surveys were limited in the central GBR as they aligned with tourism operations from Reef Magic Research Station but were included in this study as the first available data we know of on crab density in the region. All in-water surveys were conducted on reefs in green no-take or yellow conservation zones to avoid confounding fishery impacts in this assessment. Laboratory experiments for eDNA method development were conducted at Heron Island Research Station (HIRS) over multiple expeditions between October 2022 and April 2023. All collections, surveys, and experiments operated under Marine Park (G23/49210.1) and UQ Animal Ethics (2019/AE000388) permits. All statistical analyses described below were conducted in R 4.1.2 with data and code made available on figshare (67).

### Distribution of Key Cryptic Predators.

Differences in the density, distribution, and habitat associations of key rubble-dwelling predators were explored to address the hypothesis that fewer predators are present in regions where primary CoTS outbreaks are thought to originate (i.e., northern reefs; 8). Predators were surveyed in transects (120 m^2^; 4 × 30 m) placed over the reef and rubble, with 52 transects in the south (six sites; n = 8 to 12 site^−1^), six in the central GBR (two sites; n = 3 site^−1^), and 57 in the north (eight sites; n = 4 to 11 site^−1^). Transects in the north were 40 m^2^ (4 × 10 m), with all data standardized to 100 m^2^.

The primary predator of CoTS (*S. aspera*) is relatively rare and seems to prefer to live under large pieces of dead table coral (27, 38). Thus, to quantify predator density, all large rubble pieces (≥10 cm diameter) within each transect were lifted or overturned for close inspection (mean ± SE: 47 ± 3 pieces per transect; range: 4 to 136). Live corals and rubble consolidated to the substrate were not disturbed. While this method was biased toward predators living underneath large and accessible rubble pieces atop the benthos, more rigorous population surveys would be highly invasive and require extensive destruction of habitat. Previous attempts to detect these predators by breaking up rubble or with passive samplers (e.g., rubble baskets) have not been successful (27, 38, 68), so the overturning of large rubble pieces was employed here as the most effective, nondestructive, standardized technique to date that is approporiate for monitoring purposes. To refine our search effort, only the presence of the main known cryptic predators of CoTS (27)—*S. aspera*, and the Portunidae, Xanthidae, and Epialtidae (species grouped per family)—was recorded, and mean data generated. Mean (±SE) size of predators measured was 19.8 ± 0.7 mm (range: 5 to 46 mm) carapace width. Smaller decapods, expected to be more easily missed in this approach, consume considerably fewer CoTS (69, 70).

To evaluate habitat characteristics, the length and width of each overturned rubble piece were measured to determine rubble piece size (area, cm^2^), along with water depth (m), and underlying rubble bed thickness (cm) using a thin (6 mm) metal rod wiggled as far into the rubble benthos as possible. Relative benthic cover was quantified using the point-intercept method (71) with categories for live coral, dead coral, rubble, macroalgae, soft coral, sand, and other (e.g., clams and conspicuous sponges). Proportional benthic cover of each category was calculated, and mean data was determined.

Mean densities of cryptic predators were compared using a zero-inflated *glmmTMB* with a tweedie distribution to account for the high proportion of zeros (72, 73). Reef region (southern, central, northern) was used as a fixed factor with site as a random term. Separate models were developed per predator species and for total predator density. Continuous data were log-transformed for analysis and assumptions were checked and confirmed using diagnostic plots with the *DHARMa* package (74). Significant results were explored by the Tukey method using *emmeans* (75).

The extent to which physical and ecological characteristics determine the density of key cryptic CoTS predators was then explored in detail using a Bayesian SEM framework. SEMs facilitate the evaluation of a network of relationships among ecosystem variables across a range of scales (76). To construct the SEM, spatial drivers (reef, site, water depth) were used to predict responses across all levels of organization with additional parameters successively added as predictors of variables at lower levels of the ecosystem hierarchy to address structured drivers of habitat (e.g., benthic cover) and rubble microhabitat characteristics (e.g., bed thickness, piece size) on cryptic predators (*SI Appendix*, Table S1). The model focused on *S. aspera* and the Portunidae as the top predators of juvenile CoTS in rubble (27). CoTS density was included to interrogate the hypothesis that cryptic predators modulate CoTS densities. Data on adult CoTS densities were attained (CoTS Control Program; *SI Appendix*, Table S2), and an effort-normalized (i.e., observed number of CoTS divided by search time) mean annual (2012 to 2024) number of CoTS recorded per site was derived from the two locations nearest to each study site (*SI Appendix*, Table S2). As these data did not directly correspond to our empirical dataset in space or time, CoTS control data were contrast only to reef, site, and predators in this assessment (*SI Appendix*, Table S1).

The Bayesian SEM was developed using the *blavaan* package, which relies on JAGS and Stan to estimate models via Markov Chain Monte Carlo simulation (77). Weakly informative priors were specified for all fixed effects (normal distribution with mean = 0 and SD = 1, *SI Appendix*, Table S1). The model was fit with three chains and 11,000 iterations, the first 1,000 of which were discarded as burn-in. Component models were considered purely additive (*SI Appendix*, Table S1) with correlated error structures specified a priori to account for confounding variables, such as percent cover of benthic categories. Model convergence was assessed using trace, density, and autocorrelation plots and was monitored using the $\hat{R}$ convergence criterion (77). The SEM procedure produced a posterior predictive *P*-value of 0.11 with $\hat{R}$ values consistently ≤ 1.05 (*SI Appendix*, Table S1) indicating chain convergence (78). Data were standardized to scale outcomes for variables of different metrics and estimate and error values used to explore relationships among variables.

### Molecular eDNA Detection.

We developed an eDNA method to detect CoTS DNA in the gut contents of cryptic predators. First, to ensure positive detection of CoTS was viable and determine the length of time CoTS DNA can be detected in the digestive system of cryptic predators, a series of timed feeding experiments were conducted at HIRS. *S. aspera* were collected, acclimated to isolated aquarium conditions, and fed a single juvenile CoTS (<5 mm diameter). Individuals were monitored closely to detect the exact time of consumption, fixed in 100% ethanol at intervals of 1 (n = 3), 3 (n = 5), 9 (n = 2) 12 (n = 3), and 48 (n = 3) h postingestion, and frozen until use.

Suitable methods to establish sensitivity of the eDNA method were developed using a single PCR barcoding approach with CoTS-specific markers using a digital droplet assay following methods described in Uthicke et al. (79) with minor modifications, being without restriction enzyme and that droplets were generated using an automated droplet generation instrument (AutoDG, BioRad) prior to thermal cycling and droplet reading. Under sterile conditions, three main tissues of the digestional system (stomach, midgut, and abdomen) of each crab was dissected and merged for analysis. Tissue samples were placed in 1 mL (1/10 ATL, Protenise K solution 10 mg/mL) or 3 mL (1/5 ATL, Proteinase K solution 20 mg/mL, midgut only) of extraction buffer and were lysed overnight at 56 °C. DNA was extracted on a Qiacube automated extraction instrument or manually from a 600 µL subsample using DNeasy blood and Tissue kit following the manufacturer’s protocol except elution was in 3 × 50 µL TE buffer.

Two replicates (5 μL each) of these DNA extracts with 20 μL of PCR mix were subjected to droplet generation and ddPCR (conditions: 95 °C for 10 min, 1 cycle; 95 °C for 30 s, 60 °C for 1 min, 40 cycles; 98 °C for 10 min, 1 cycle; 10 °C infinite hold) as described in ref. 79. Data were reported as copies per sample. Because eDNA can be prone to contamination, a rigorous quality control workflow was established to ensure the entire laboratory workflow remained contamination free. This included negative controls at all levels of sample handling. Positive controls were also included for each batch of extractions. All field and laboratory controls returned no presence of CoTS DNA, so positive detection was defined as a sample with one or more positive droplets in ddPCR assay.

Once best-practice eDNA extraction and detection methods were established, wild-type predators were collected to investigate this predator–prey interaction in nature. Collections were conducted in March when early-stage juvenile CoTS are expected to be available in rubble (37) prior to transitioning to coral-feeding at ~5 to 7 mo postsettlement and ~8 mm juvenile diameter (80). In the southern GBR (March 2023), *S. aspera* (n = 17) were collected and fixed immediately in 100% ethanol. Five animals were used in methods testing, with two tissues from the remaining 12 analyzed with ddPCR assay (79), as above. In the northern GBR (March 2024), 63 specimens comprising 21 species of decapod (*SI Appendix*, Table S3) known to consume juvenile CoTS (27), were collected and three tissues analyzed in the same way. No specimens were retained from the central GBR.

### Scalable Impact of Cryptic Predators.

The impact of cryptic predators on CoTS populations was extrapolated in two ways. First, daily rates of potential predation were calculated using mean predator density (this study) and published rates of juvenile consumption by *S. aspera* (5.8 ind. d^−1^) and the Portunidae (0.4 ind. d^−1^) from mesocosm experiments that mimicked rubble habitat complexity with alternate prey (27). Predation rates of portunids were used for xanthids and epialtids as the best available data. Community-level estimates were calculated for each transect and averaged per region. Then, daily rates of realized predation were estimated based on the density of wild-type specimens found with CoTS DNA in the southern and northern GBR. Timed feeding trials (as above) allowed us to estimate a period of consumption based on equivalent DNA copy numbers using the following equation to convert the proportion of positive DNA detections in wild-type specimens to an estimated feeding rate:

$$RP=\frac{\left(D\times pDNA\right)}{d},$$

where *RP* is the daily rate of realized predation, *D* is the density of decapods measured in transects, *pDNA* is the proportion of the wild population detected with CoTS DNA, and *d* is the number of days (or assumed digestion window) based on equivalent copy number detection in timed feeding trials (24 or 48 h; Fig. 3 *A*–*C*). Mean data per region were used in calculations with error estimated using Bernoulli probability of success with *binom.test* (81). This calculation assumed that one juvenile was consumed in full per predator.

## Supplementary Material

Appendix 01 (PDF)

## Data Availability

The authors declare that all relevant data (Excel spreadsheets) and code (R files) are available on figshare (https://doi.org/10.6084/m9.figshare.28267874) (67).
