# Supplementary material for: eDNA confirms lower trophic interactions help to modulate population outbreaks of the notorious crown-of-thorns sea star
Source: Proc Natl Acad Sci U S A. 2025 Mar 10;122(11):e2424560122. doi: 10.1073/pnas.2424560122 (PMC11929471; doi:10.1073/pnas.2424560122)
Supplement: Supplementary file 1 — Appendix 01 (PDF) [file pnas.2424560122.sapp.pdf]

Supplementary Material for

eDNA confirms lower trophic interactions help to modulate population outbreaks of the notorious Crown-of-Thorns Sea star

Kennedy Wolfe[a,b,1], Amelia A. Desbiens[a,c], Frances Patel[d], Sarah Kwong[d,e], Eric Fisher[f], Peter J. Mumby[a], Sven Uthicke[d]

a. School of the Environment, The University of Queensland, Brisbane QLD 4072
b. Marine Biology and Ecology Consultant, Geeveston, TAS 7116, Australia
c. CSIRO Environment, Brisbane QLD 4072
d. Australian Institute of Marine Science, Townsville QLD 4810
e. James Cook University, Townsville QLD 4811
f. GBR Biology, Experience Co, Cairns QLD 4870

1. Corresponding author details:

Kennedy Wolfe

Email: k.wolfe90@yahoo.com

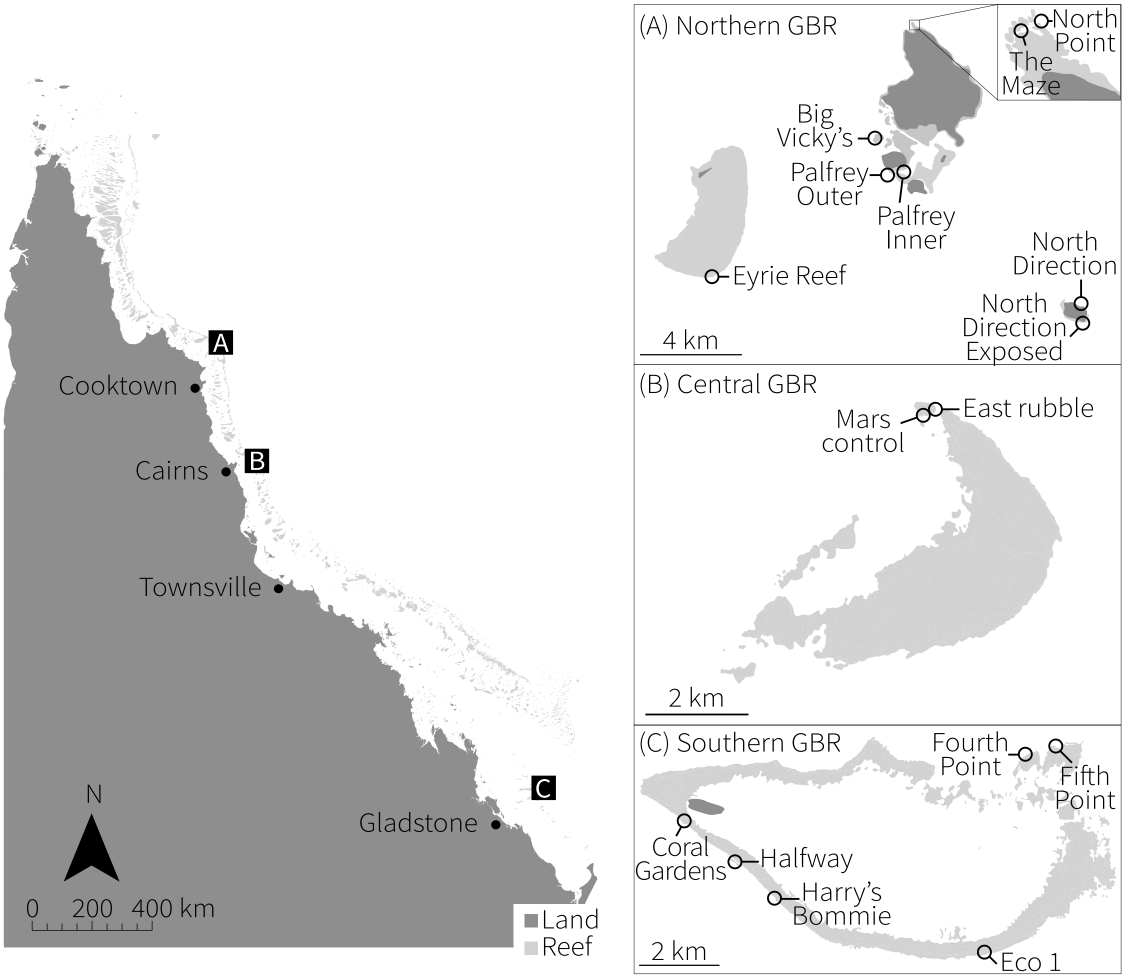

Figure S1: Indication of sites visited in this project in the northern (Lizard Island; n = 57 transects; n = 4–11 $site^{-1}$), central (Moore Reef; n = 6 transects; two sites; n = 3 $site^{-1}$) and southern (Heron Island; n = 52 transects; n = 8–12 $site^{-1}$) GBR, Australia.

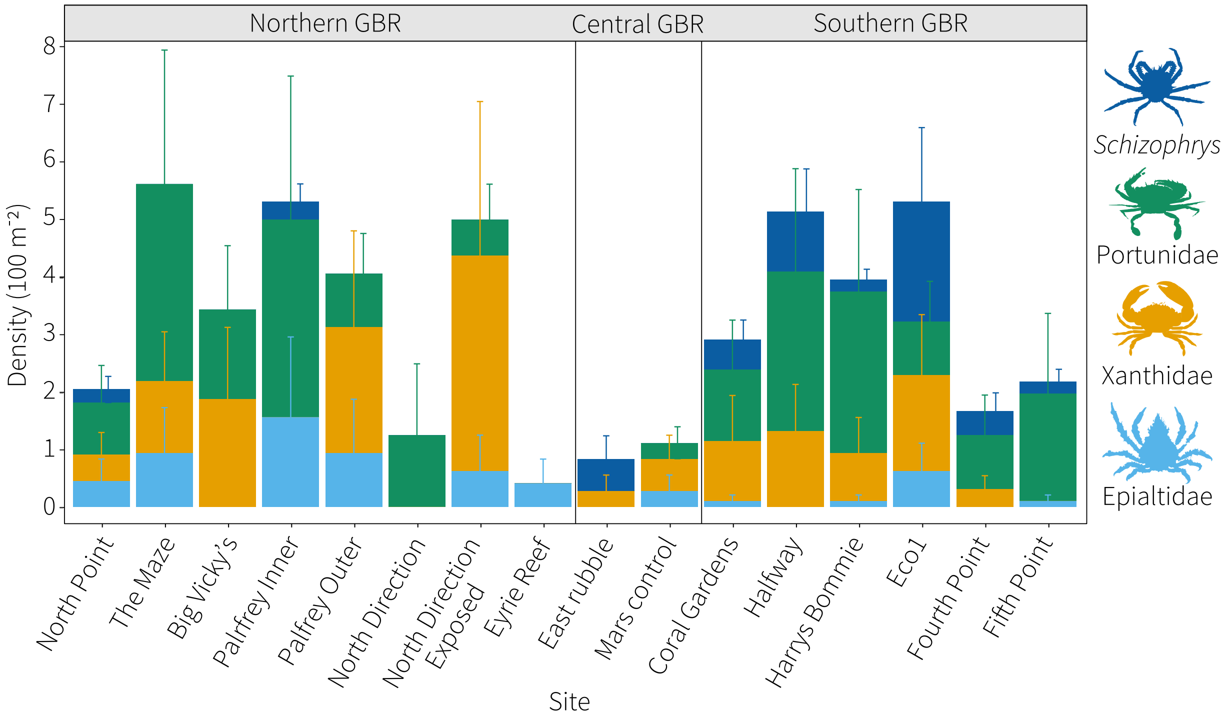

Figure S2: Mean (±SE) density of key predators of juvenile CoTS in rubble among survey sites in the northern, central and southern Great Barrier Reef.

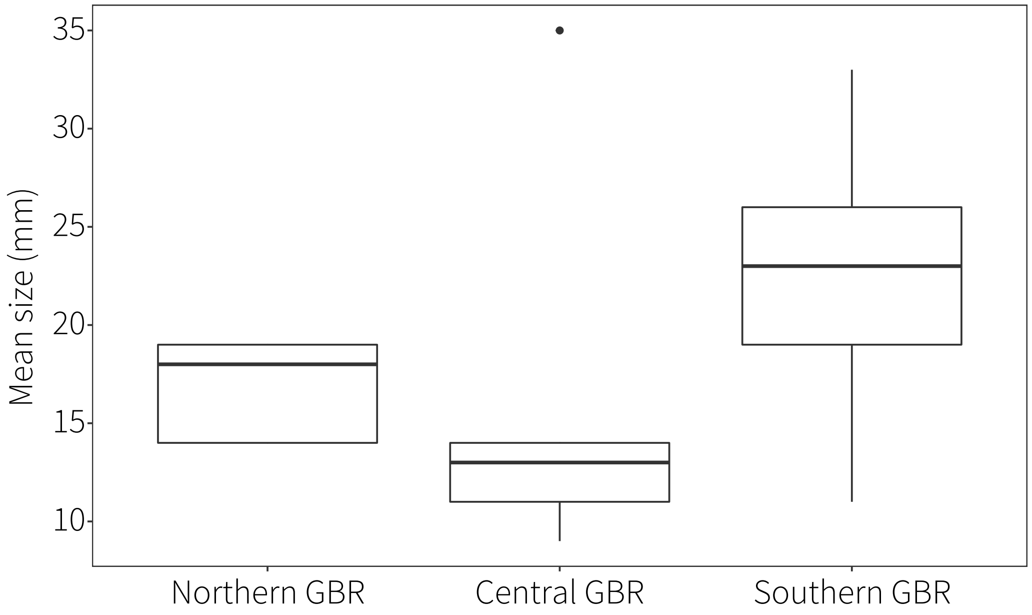

Figure S3: Mean carapace width (mm) of *Schizophrys aspera* across transects and collections in the northern (n=5), central (n=6) and southern (n=41) GBR. Black point represents one large male *Schizophrys* sp. found at Moore Reef.

Table S1: Component model specification for scaled variables in the Bayesian structural equation model including $\hat{R}$ scores.

Response	Fixed effects	$\hat{R}$
Rubble cover	Reef + Site + Depth	1.00
Sand cover	Reef + Site + Depth	1.00
Live coral cover	Reef + Site + Depth	1.00
Hard substrate cover	Reef + Site + Depth	1.00
Rubble bed thickness	Reef + Site + Depth + Rubble + Sand + Live coral + Hard substrate	1.00
Rubble piece size	Reef + Site + Depth + Rubble + Sand + Live coral + Hard substrate	1.00
Number of rubble pieces	Reef + Site + Depth + Rubble + Sand + Live coral + Hard substrate	1.00
Density of *S. aspera*	Reef + Site + Depth + Rubble + Sand + Live coral + Hard substrate + Bed thickness + Piece size + Number of pieces	1.00
Density of Portunidae	Reef + Site + Depth + Rubble + Sand + Live coral + Hard substrate + Bed thickness + Piece size + Number of pieces	1.00
Density of adult CoTS	Reef + Site + *S. aspera* + Portunidae	1.00

Table S2: Effort-normalised density (i.e., number of CoTS observed divided by search time) of CoTS derived from Control Program.

		Control Site 1		Control Site 2		CoTS density (per unit effort)		
Reef	Site	Latitude	Longitude	Latitude	Longitude	Site 1	Site 2	Mean
Lizard Island	North Point	145.4547	-14.6451	145.4511	-14.6465	0.022	0.005	0.013
	The Maze	145.4547	-14.6451	145.4536	-14.6463	0.022	0.064	0.043
	Big Vicky's	145.4372	-14.6826	145.4404	-14.6837	0.101	0.319	0.210
	Palfrey Inner	145.4472	-14.6991	145.4426	-14.696	0.035	0.031	0.033
	Palfrey Outer	145.4472	-14.6991	145.4426	-14.696	0.035	0.031	0.033
	Nth Direction	145.5127	-14.7428	145.5117	-14.7419	0.301	0.010	0.156
	Nth Direction Exposed	145.5127	-14.7428	145.5059	-14.7436	0.301	0.197	0.249
	Eyrie Reef	145.3796	-14.7338	145.3845	-14.7318	0.252	0.382	0.317
Moore Reef	East rubble	146.2279	-16.8478	146.231	-16.8454	0.915	0.004	0.459
	Mars control	146.2279	-16.8478	146.231	-16.8454	0.915	0.004	0.459
Heron Island	Coral Gardens	151.9139	-23.4477	151.918	-23.4503	0.013	0.008	0.011
	Halfway	151.918	-23.4503	151.9219	-23.4529	0.008	0.015	0.012
	Harry's Bommie	151.9256	-23.4557	151.9292	-23.4588	0.027	0.127	0.077
	Eco 1	151.965	-23.4722	151.97	-23.4724	0.029	0.012	0.021
	Fourth Point	151.984	-23.4359	151.9872	-23.4327	0.174	0.109	0.141
	Fifth Point	151.9901	-23.4347	151.992	-23.4314	0.113	0.299	0.206

Table S3: Invertebrate species collected during surveys in the southern (Heron Island, March 2023) and northern (Lizard Island, March 2024) GBR for eDNA analysis to detect presence of CoTS DNA. Green cells denote positive detection; sites outlined in Figure S1.

**Site**	**Order**	**Family**	**Species**	**CoTS DNA detection**
Heron Island				
Eco 1	*Decapoda*	*Majidae*	*Schizophrys aspera*	-
Eco 1	*Decapoda*	*Majidae*	*Schizophrys aspera*	+
Eco 1	*Decapoda*	*Majidae*	*Schizophrys aspera*	-
Eco 1	*Decapoda*	*Majidae*	*Schizophrys aspera*	-
Eco 1	*Decapoda*	*Majidae*	*Schizophrys aspera*	+
Eco 1	*Decapoda*	*Majidae*	*Schizophrys aspera*	-
Eco 1	*Decapoda*	*Majidae*	*Schizophrys aspera*	-
Fourth Point	*Decapoda*	*Majidae*	*Schizophrys aspera*	-
Fourth Point	*Decapoda*	*Majidae*	*Schizophrys aspera*	-
Halfway	*Decapoda*	*Majidae*	*Schizophrys aspera*	-
Halfway	*Decapoda*	*Majidae*	*Schizophrys aspera*	-
Halfway	*Decapoda*	*Majidae*	*Schizophrys aspera*	-
Lizard Island				
Palfrey Inner	*Decapoda*	*Xanthidae*	*Actaeodes hirsutissimus*	-
Big Vicky's	*Decapoda*	*Xanthidae*	*Atergatis floridus*	-
North Point	*Decapoda*	*Xanthidae*	*Atergatis floridus*	-
Palfrey Inner	*Decapoda*	*Xanthidae*	*Atergatis floridus*	-
Palfrey Inner	*Decapoda*	*Xanthidae*	*Atergatis floridus*	-
Palfrey Outer	*Decapoda*	*Inachiae*	*Camposcia retusa*	-
Eyrie Reef	*Decapoda*	*Xanthidae*	*Chlorodiella nigra*	-
Big Vicky's	*Decapoda*	*Xanthidae*	*Cyclodius ungulatus*	-
North Point	*Decapoda*	*Xanthidae*	*Cyclodius ungulatus*	-
Big Vicky's	*Decapoda*	*Xanthidae*	*Cyclodius ungulatus*	+
Big Vicky's	*Decapoda*	*Xanthidae*	*Cyclodius ungulatus*	-
Palfrey Inner	*Decapoda*	*Xanthidae*	*Cyclodius ungulatus*	-
Palfrey Inner	*Decapoda*	*Xanthidae*	*Cyclodius ungulatus*	-
Palfrey Inner	*Decapoda*	*Xanthidae*	*Cyclodius ungulatus*	-
Big Vicky's	*Decapoda*	*Xanthidae*	*Etisus sp.*	-
Big Vicky's	*Decapoda*	*Xanthidae*	*Etisus sp.*	-
North Point	*Decapoda*	*Xanthidae*	*Etisus anaglyptys*	-
Big Vicky's	*Decapoda*	*Pilumnidae*	*Heteropilumnus*	-
North Point	*Decapoda*	*Xanthidae*	*Paractaea rufopunctata*	-
Palfrey Inner	*Decapoda*	*Majidae*	*Schizophrys aspera*	-
North Point	*Decapoda*	*Majidae*	*Schizophrys aspera*	-
North Point	*Decapoda*	*Majidae*	*Schizophrys aspera*	-
North Point	*Decapoda*	*Majidae*	*Schizophrys aspera*	-
Palfrey Inner	*Decapoda*	*Majidae*	*Schizophrys aspera*	-
North Point	*Decapoda*	*Xanthidae*	*Soliella flava*	-

North Point	*Decapoda*	*Xanthidae*	*Soliella flava*	+
North Direction	*Decapoda*	*Portunidae*	*Thalamita admete*	-
North Point	*Decapoda*	*Portunidae*	*Thalamita admete*	-
Palfrey Inner	*Decapoda*	*Portunidae*	*Thalamita admete*	-
Palfrey Inner	*Decapoda*	*Portunidae*	*Thalamita admete*	-
Palfrey Inner	*Decapoda*	*Portunidae*	*Thalamita admete*	-
Palfrey Inner	*Decapoda*	*Portunidae*	*Thalamita admete*	+
Big Vicky's	*Decapoda*	*Portunidae*	*Thalamita bouvieri*	+
North Point	*Decapoda*	*Portunidae*	*Thalamita coeruleipes*	+
North Point	*Decapoda*	*Portunidae*	*Thalamita coeruleipes*	+
Big Vicky's	*Decapoda*	*Portunidae*	*Thalamita coeruleipes*	-
North Point	*Decapoda*	*Portunidae*	*Thalamita coeruleipes*	-
North Point	*Decapoda*	*Portunidae*	*Thalamita coeruleipes*	-
North Point	*Decapoda*	*Portunidae*	*Thalamita coeruleipes*	-
North Point	*Decapoda*	*Portunidae*	*Thalamita coeruleipes*	-
Big Vicky's	*Decapoda*	*Portunidae*	*Thalamita pelsarti*	-
North Point	*Decapoda*	*Portunidae*	*Thalamita pelsarti*	-
Palfrey Inner	*Decapoda*	*Portunidae*	*Thalamita pelsarti*	-
Palfrey Inner	*Decapoda*	*Portunidae*	*Thalamita pelsarti*	-
Palfrey Inner	*Decapoda*	*Portunidae*	*Thalamita pelsarti*	-
Palfrey Inner	*Decapoda*	*Portunidae*	*Thalamita pelsarti*	-
North Point	*Decapoda*	*Portunidae*	*Thalamita prymna*	-
North Direction Exposed	*Decapoda*	*Portunidae*	*Thalamita prymna*	-
North Point	*Decapoda*	*Portunidae*	*Thalamita prymna*	-
North Point	*Decapoda*	*Portunidae*	*Thalamita sp.*	-
North Point	*Decapoda*	*Portunidae*	*Thalamita sp.*	-
North Direction Exposed	*Decapoda*	*Portunidae*	*Thalamitoides quadridens*	-
Palfrey Inner	*Decapoda*	*Portunidae*	*Thalamitoides tridens*	-
Eyrie Reef	*Decapoda*	*Epialtidae*	*Tiarinia cornigera*	-
North Point	*Decapoda*	*Epialtidae*	*Tiarinia cornigera*	-
North Point	*Decapoda*	*Epialtidae*	*Tiarinia cornigera*	-
North Point	*Decapoda*	*Epialtidae*	*Tiarinia cornigera*	+
Palfrey Inner	*Decapoda*	*Epialtidae*	*Tiarinia cornigera*	-
Palfrey Inner	*Decapoda*	*Epialtidae*	*Tiarinia cornigera*	-
Palfrey Inner	*Decapoda*	*Epialtidae*	*Tiarinia cornigera*	-
Palfrey Inner	*Decapoda*	*Epialtidae*	*Tiarinia cornigera*	-
Palfrey Inner	*Decapoda*	*Epialtidae*	*Tiarinia cornigera*	-
Palfrey Inner	*Decapoda*	*Epialtidae*	*Tiarinia cornigera*	-
